# Survey and analysis of the current state of residency training in medical-school-affiliated hospitals in China

**DOI:** 10.1186/1472-6920-14-111

**Published:** 2014-06-02

**Authors:** Hong Bo, Dong-Hua Zhang, Tian-Ming Zuo, Dong-Bo Xue, Jin-Song Guo, Mei-Na Liu, Jing-Zhu Dong, Bao-Zhi Sun, Jin Zhou

**Affiliations:** 1 The First Affiliated Hospital, Harbin Medical University, Harbin, China; 2 Medical Center of Research and Development, China Medical University, Shenyang, China; 3 Department of Biostatistics, College of Public Health, Harbin Medical University, Harbin, China

**Keywords:** Residency training, Global standards for postgraduate medical education, Medical-school-affiliated hospitals, China

## Abstract

**Background:**

Since the global standards for postgraduate medical education (PGME) were published in January 2003, they have gained worldwide attention. The current state of residency training programs in medical-school-affiliated hospitals throughout China was assessed in this study.

**Methods:**

Based on the internationally recognized global standards for PGME, residents undergoing residency training at that time and the relevant residency training instructors and management personnel from 15 medical-school-affiliated hospitals throughout China were recruited and surveyed regarding the current state of residency training programs. A total of 938 questionnaire surveys were distributed between June 30, 2006 and July 30, 2006; of 892 surveys collected, 841 were valid.

**Results:**

For six items, the total proportions of “basically meets standards” and “completely meets standards” were <70% for the basic standards. These items were identified in the fields of “training settings and educational resources”, “evaluation of training process”, and “trainees”. In all fields other than “continuous updates”, the average scores of the western regions were significantly lower than those of the eastern regions for both the basic and target standards. Specifically, the average scores for the basic standards on as many as 25 of the 38 items in the nine fields were significantly lower in the western regions. There were significant differences in the basic standards scores on 13 of the 38 items among trainees, instructors, and managers.

**Conclusions:**

The residency training programs have achieved satisfactory outcomes in the hospitals affiliated with various medical schools in China. However, overall, the programs remain inadequate in certain areas. For the governments, organizations, and institutions responsible for PGME, such global standards for PGME are a very useful self-assessment tool and can help identify problems, promote reform, and ultimately standardize PGME.

## Background

Medical education is a continuous, lifelong educational system that includes basic medical school education, postgraduate education, and continuing education [1]. Postgraduate medical education (PGME) is an important part of the continuing medical education process and takes place after the completion of medical school; its purpose is to cultivate the ability to work both independently and under the guidance of instructors [2]. PGME is a key element of the training of medical professionals, and the quality of PGME directly affects the quality of clinical medical personnel training.

Currently, PGME remains a significant issue in global medical education and global health because of pipeline/capacity issues and quality issues related to providers. The global standards for PGME are one of three sets of global standards for medical education (i.e., global standards for undergraduate medical education, global standards for PGME, and global standards for continuing medical education) [3]. These standards were developed and published in January 2003 by the project group appointed by the World Federation for Medical Education and are primarily used by governments, organizations, and institutions in charge of PGME to develop a new framework for self-assessment [4]. The purpose of the standards is to promote reform and ultimately standardize PGME. In addition, because the standards are internationally recognized, they provide a basis for the evaluation and accreditation of PGME programs by various countries and regions.

PGME started late in China and has been gradually systemized and standardized since 1993 [5]. To maintain pace with international trends in higher medical education, the Ministry of Health established the PGME committee in December 2005. The main duties of the committee are to guide, coordinate, and manage national PGME; to conduct research on national policies for PGME; and to develop and implement a national PGME program and management approach under the leadership of the Ministry of Health [6]. To date, PGME in China has undergone more than ten years of exploration and development and has achieved significant outcomes. However, due to various factors, PGME remains the weakest link in the entire higher medical education system. As a country with a large population, China continues to face important public health challenges. With the rapid development of science and technology and acceleration of globalization, it is urgent that China improve medical education to meet international standards.

At present, PGME in China primarily consists of standardized residency training, standardized training of general practitioners, and standardized specialist training [7]. The present study utilized global standards [8-10] for PGME in a questionnaire survey regarding the current state of residency training among medical-school-affiliated hospitals throughout China, with the goal of identifying problems, proposing reform measures, supporting improvements, and promoting standardization and internationalization for residency training in China.

## Method

### Survey period and subjects

In 2006, there were approximately 120 medical schools in China. Of these, one medical school was randomly selected from each geographic region, so that 12 medical schools were chosen overall. Altogether, 64 hospitals were affiliated with the 12 medical schools. Among these, 49 were grade-A, class-three hospitals and qualified to provide residency training. Of the 49 affiliated hospitals, one or two were randomly selected from each of the 12 medical schools. Ultimately, a total of nine hospitals in the eastern region of China and six hospitals in the western region of China were included in this study. The nine hospitals in the eastern region of China included the following: Xuanwu Hospital of Capital University of Medical Sciences, the First and Second Affiliated Hospitals of China Medical University, Jiangsu Provincial People's Hospital of Nanjing Medical University, the Second Affiliated Hospital of Hebei Medical University, the Second Affiliated Hospital of Dalian Medical University, the Affiliated Hospital of Tianjin Armed Police Medical School, and the First and Second Affiliated Hospitals of Harbin Medical University. The six hospitals in the western region of China included the following: the First Affiliated Hospital of Chongqing Medical University, the Affiliated Hospital of Medical College of Xinjiang Shihezi University, the First and Second Affiliated Hospitals of Shanxi Medical University, the Affiliated Hospital of Inner Mongolia Medical College, and the Affiliated Hospital of the Medical College of Qinghai University. Between June 30, 2006 and July 30, 2006, a questionnaire survey was conducted among all residents undergoing residency training at that time and all the relevant residency training instructors and management personnel.

Approval for this study was obtained from the Committee for Medical Research Ethics of the First Affiliated Hospital of Harbin Medical University, the authority on research ethics in China.

### Survey method and survey instrument

In the preface part of the questionnaire, there is a brief introduction on PGME and “the global standards for PGME”, a table about the basic information of respondents and an instruction about how to complete the questionnaire. The main part of the questionnaire is a Chinese version of the WFME global standards of the PGME [11], including 38 items in nine fields (see Results below for details). Each item in the questionnaire was further divided into two levels, one level for trainers who met the basic requirements (basic standard) and another level for those who met the requirements for outstanding qualifications (target standard).

To describe the answers to each item in the survey, scores of 1, 2, 3, or 4 were assigned, corresponding to “does not meet standards”, “partially meets standards”, “basically meets standards”, and “completely meets standards”. The respondents could give their answer in the parentheses which exist just behind both basic standard and target standard of each item. The average score for each field was calculated based on scores for each item and was used to evaluate the overall survey results for the nine fields.

To improve the content validity of survey instrument, the survey instrument was pilot tested among 3 trainees, 3 instructors, and 3 management personnel who were not participating in the current study. The respondents made evaluations on questionnaire format and understandability of the questions from their respective point of view, and according to which, we revised the questionnaire.

### Data processing and statistical analysis

The SAS 9.13 software package was used for data entry and statistical analysis. Student's t-test and mean variance analysis were used for two-group and three-group comparisons, respectively. P < 0.05 was considered statistically significant.

## Results

The on-site survey was conducted among 15 randomly selected affiliated hospitals of the 12 medical schools. A total of 938 questionnaires were distributed, and 892 were collected, a recovery rate of 95.1%. Of the surveys collected, 841 were valid, while the remaining 51 were invalid owing to incomplete data, resulting in a validity rate of 94.3%. The respondent demographics are shown in Table 1.

**Table 1:** Respondent demographics

**Characteristics**	**No.**	**%**
Overall	841	100
**Age, y**		
24-29	270	32.1
30-39	255	30.3
40-49	229	27.2
50-59	63	7.5
60-68	24	2.9
**Sex**		
Male	440	52.3
Female	401	47.7
**Degree**		
Doctor	83	9.9
Master	274	32.6
Bachelor	484	57.5
**Group**		
Trainees	342	40.7
Instructors	247	29.4
Managers	252	29.9
**Subject**		
Internal medicine	327	38.9
Surgery	259	30.8
Gynecology	58	6.9
Pediatrics	46	5.5
Others*	151	17.9
**Geographic region**		
Eastern region of China	500	59.5
Western region of China	341	40.5

Others* includes 22 managers who responsible for overall management, can not be divided into any subject.

### General overview of the current state of residency training in medical-school-affiliated hospitals in China, based on global standards for PGME

**Figure 1:**
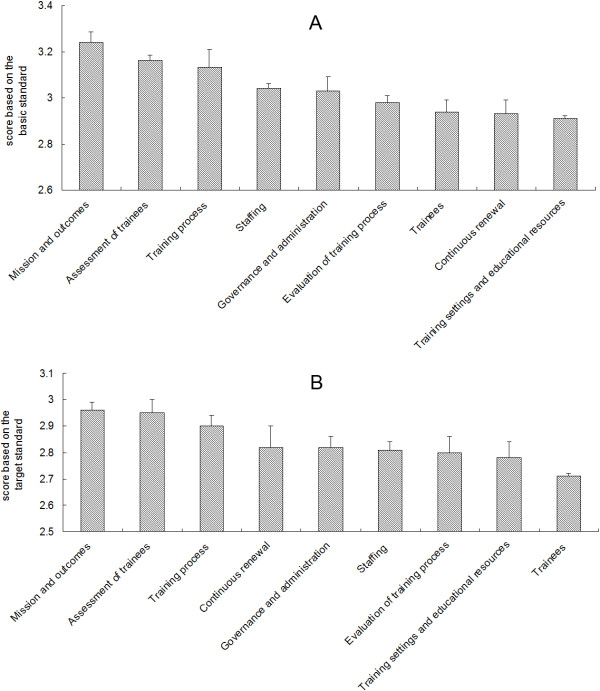
**Overall survey results for the nine fields of the global standards for postgraduate medical education. A**: Scores based on the basic standards. **B**: Scores based on the target standards.

Overall survey results for the nine fields of the global standards for PGME were shown in Figure 1. Based on the items’ scores for basic standards (Figure 1A), the nine fields were ranked as follows (in descending order):

1. Mission and outcomes: professionalism and autonomy; statements of mission and outcomes; training outcomes; and participation in the formulation of mission and outcomes.

2. Assessment of trainees: relationship between assessment and training; assessment methods; and feedback to trainees.

3. Training process: scientific methods; learning approaches; relationship between training and service; management of training; training structure, composition and duration; and training content.

4. Staffing: obligations and development of trainers; and appointment policy.

5. Governance and administration: professional leadership; governance; administration; requirements and regulations; and funding and resource allocation.

6. Evaluation of training process: authorization and monitoring of training settings; using trainee performance; feedback from trainers and trainees; mechanisms for program evaluation; and involvement of stakeholders.

7. Trainees: number of trainees; admission policy and selection; working conditions; support and counselling for trainees; and trainee representation.

8. Continuous renewal.

9. Training settings and educational resources: clinical settings and patients; clinical teams; physical facilities and equipment; research; information technology; educational expertise; and training in other settings and abroad.

The survey results for the 38 items in the nine fields are shown in Table 2. For six items, the total proportions of “basically meets standards” and “completely meets standards” were <70% for the basic standards. These items were identified in the fields of “training settings and educational resources” (information technology, 69.4%; educational expertise, 62.4%; training in other settings and abroad, 53.4%), “evaluation of training process” (mechanisms for program evaluation, 65%; involvement of stakeholders, 68.9%), and “trainees” (trainee representation, 61.4%).

**Table 2:** The survey results of the 38 items in the 9 domains [median proportions (Inter-Quartile Range)]

	**Does not meet standards (%)**	**Partially meets standards (%)**	**Basically meets standards (%)**	**Completely meets standards (%)**
Basic standards	0.5 (0.2-11.1)	19.05 (7.1-29.3)	49.45 (40-60.9)	28.4 (21.4-42.3)
Target standards	4.15 (1.8-14.2)	25.45 (11.6-35.3)	49.0 (42.6-56.9)	18.75 (14.3-25.6)

### Comparison of the survey answers between eastern and western regions

**Figure 2:**
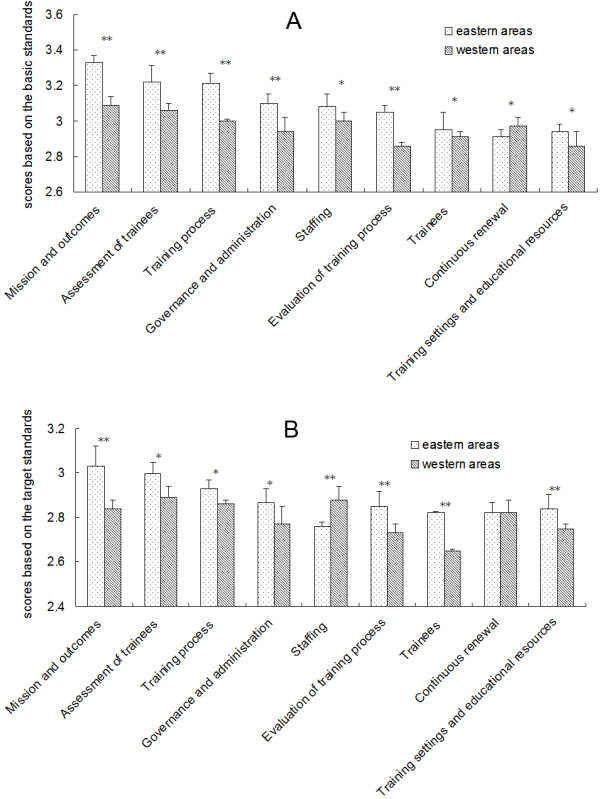
**Comparison of the survey results for the nine fields of the global standards for postgraduate medical education among different regions. A**: Scores based on the basic standards. **B**: Scores based on the target standards. Significance level: *P < 0.05 **P < 0.001.

A comparison of survey responses indicates significant differences between the eastern and western regions with respect to the current state of residency training in medical-school-affiliated hospitals. In all fields other than “continuous updates”, the average scores of the economically backward western regions were significantly lower than those of the economically developed eastern regions for both the basic and target standards (Figure 2). Specifically, the average scores for the basic standards of up to 25 of the 38 items in the nine fields were significantly lower in the western regions.

### Comparison of trainees, instructors, and management personnel

**Figure 3:**
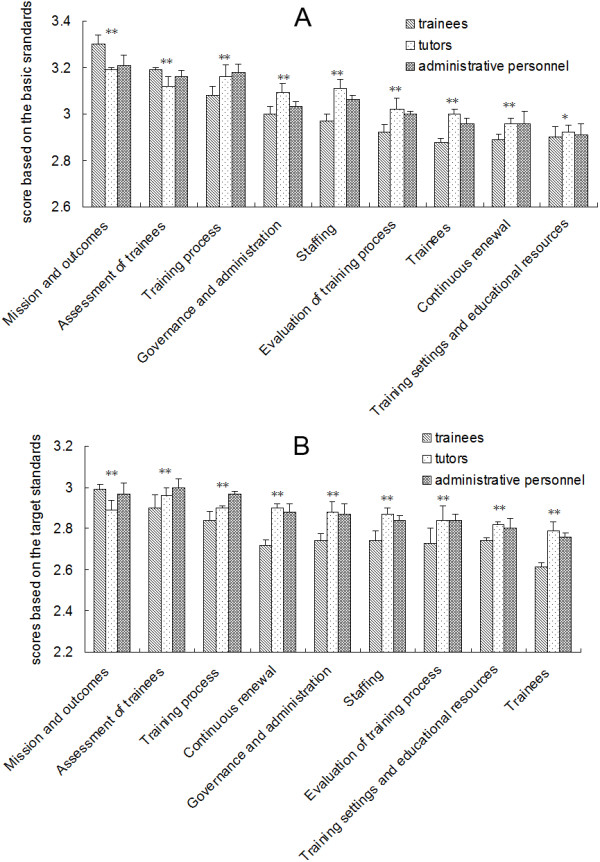
**Comparison of the survey results for the nine fields of the global standards for postgraduate medical education among different personnel categories. A**: Scores based on the basic standards. **B**: Scores based on the target standards. Significance level: *P < 0.05 **P < 0.001.

To determine whether there were differences in understanding regarding the global standards for PGME among various personnel, the survey responses of trainees, trainers, and management personnel were compared (Figure 3). There were significant differences in the scores on basic standards for 13 of the 38 items among the three personnel groups. The 13 items were primarily from the following six fields: “mission and outcomes” (professionalism and autonomy, training outcomes), “training process” (learning approaches, training content, and management of training), “trainees” (admission policy and selection, trainee representation), “training settings and educational resources” (training in other settings and abroad), “evaluation of the training process” (mechanisms for program evaluation, feedback from trainers and trainees, and using trainee performance), and governance and administration (funding and resource allocation, requirements and regulations).

## Discussion

Since the global standards for PGME were published in January 2003 [11], they have gained worldwide attention. As internationally recognized standards, they play an important role in improving and enhancing the quality of medical education worldwide.

**Figure 4:**
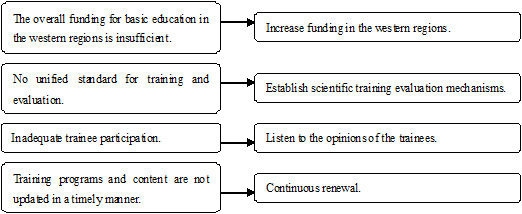
Key challenges and recommended strategies.

In recent years, with the development of the socio-economy, increased foreign exchange, and health care reform in China, medical education has undergone reform and development. Improving medical education in China to meet international standards has become an urgent goal; at the same time, the establishment of global standards for medical education has significantly guided the promotion of standardization and internationalization of medical education in China. This study utilized the global standards for PGME to conduct an on-site survey among participating residents of 15 medical-school-affiliated hospitals and subsequently performed an in-depth analysis of existing problems within the training system.Since 1993, these 15 hospitals have developed residency training, gained the attention of leaders at all levels, developed related regulations, introduced supporting policies, and gradually standardized their training in a scientific way. The survey results show that satisfactory outcomes have been achieved in the following five fields: “mission and outcomes”, “assessment of trainees”, “training process”, “staffing”, and “governance and administration”. The outcomes in these fields provide a strong foundation to ensure the quality of training. However, outcomes in the other four fields are lagging and require further improvement. Our data begins to characterize the following problems and suggest several strategies (Figure 4) to address them, although more data are needed to add to this initial work.

First, we believe that overall funding for education in the western regions is insufficient; compared with funding in the eastern regions and in China as a whole, there is a large gap. Regional economic factors in China create an imbalance in education funding between the eastern and western regions of the country. Of the western regions, economic strength is relatively weak and development is more strongly affected by some external factors, such as regional limitations; consequently, education funding has been inadequate. The 2004 data for overall funding of education shows in the case that the numbers of students receiving on-campus education (Kindergarten, elementary school, junior high school, senior high school, institution of higher learning) per one hundred thousand populations were 20063 in the western regions and 19379 in the eastern regions, the total funding for education in the western regions was ¥132,313.721 million, which was only 21.31% of overall state funding for education in China; while funding in the eastern regions was ¥337,221.838 million, which was 54.32% of overall state funding for education, and ¥204,908.117 million higher than the funding level of the western regions. Data from 2004 shows in the case that the numbers of students receiving basic education (Kindergarten, elementary school, junior high school) per one hundred thousand population were 16625 in the western regions and 13881 in the eastern regions, the total funding for basic education in the western regions was ¥95,411.72 million, which was only 23.07% of overall state funding for basic education in China; in contrast, 52.41% of overall state funding for basic education was allotted to the eastern regions, an amount equal to ¥121,283.904 million more than that provided to the western regions. These data indicate a large discrepancy in funding for education between the western and eastern regions. Additionally, these data document a serious shortage of funding for education in the western regions. Although we could not obtain the accurate data on the funding for medical education in the western and eastern regions, the data on “general education” and “basic education” funding could, to some extent, reflect the amount of medical education funding. The lack of funding for medical education has slowed the development of the training base for postgraduate education, the launching of training programs, and the development of faculty resources.

To address this problem, all levels of government and medical institutions must increase their funding, set aside special funds for PGME dedicated to the construction of the training base, and support various training programs for PGME to meet the increasing needs of these programs. The western regions, with their weak economic foundations, should devote greater efforts to promoting the comprehensive and coordinated development of medical education in different areas. The training base must also raise funds through various channels to establish the needed training funding, and trainees themselves should bear some of these costs. Various funds should be used for specified purposes and monitored under strict guidelines.

Second, our results show that there is no unified standard for training and evaluation. Assessment mechanisms are inadequate, affecting the quality of training. Currently, there is no systematic, comprehensive assessment index system for standardized training. The Ministry of Health has issued documents regarding PGME, but the description of the standards for training and assessment is very general and has no specific quantitative measurement system; therefore, there is significant room for discretion in practice, making assessments of clinical skills less robust.

The lack of a unified standard for training and evaluation results in inconsistent training quality, a problem that the statistical results of the questionnaire alone are not sufficient to explain. Without objective assessment measures, it is difficult to compare the advantages and disadvantages of different training programs, making it challenging to improve training methods.

To address this problem, government authorities should establish special assessment agencies to create scientific, standardized, and realistic evaluation mechanisms. A unified standard evaluation system should be developed. Assessment criteria and requirements should be clarified for the training base, the training process, the trainees, the instructors, and the quality of training. In addition, government authorities should explore strict evaluation methods and conduct periodic evaluations. Government authorities should also pay attention to feedback to humanize management and exert greater effort toward developing a fair, just, open, scientific, and effective evaluation system.

Third, we believe that trainee participation is inadequate. Trainees are rarely involved in the development of the various rules and regulations related to training or issues such as the structure of training programs, the design of work conditions, or the evaluation process. The vast majority of medical-school-affiliated hospitals have established advisory bodies to provide support, advice, and guidance for the trainees but have often overlooked the ideas of the trainees themselves, such as their purposes, motivations, aspirations, demands, degrees of understanding, interests, views, and confidence levels. Therefore, during the entire training process, trainees are merely passive recipients of knowledge and skills, a situation that directly affects the trainees’ attitudes and eventually the quality of training.

The results of this study show that different personnel, including trainees, instructors, and management personnel, may differ significantly in their awareness of global standards for PGME due to their differing starting points or perspectives. These differences highlight the need to view issues from multiple angles and to better understand trainees’ ideas and requirements [12]. Therefore, the authorities in charge of training should encourage trainee representatives to actively participate in the planning and development of training programs, training conditions, assessment processes, and training-related rules and regulations. Additionally, they should mobilize trainee initiatives.

Fourth, the training programs and their contents were not updated in a timely manner. Updates of training programs, content, and methods occur slowly and are often not targeted or practical; furthermore, the training does not always meet the personal development needs and requirements of trainees. Moreover, when the proportion of trainees in different disciplines changes significantly, some educational resources, including the composition of training instructors, training conditions, and training facilities, are not adjusted accordingly in a timely manner.

Residency training is a dynamic process, and training plans, objectives, and outcomes should adapt to the progress of society, the national economy, the development of medical science, and the needs of the population in general. Government authorities should establish a special institution to conduct periodic reviews of training objectives, training programs and content, training conditions, training methods, and assessment criteria and make the appropriate changes and adjustments based on identified needs. Such measures will enable residency training to keep pace with the times [13,14].

There remain some problems with the implementation of residency training in the medical-school-affiliated hospitals in China, but we believe that international and inter-school exchanges and learning will promote the process of residency training internationalization and continue to enhance PGME in China.

Our study is subject to several limitations. First, the representativeness of the data is limited. The 15 hospitals were not randomly selected from all hospitals qualified to provide residency training in China but were all grade-A, class-three hospitals selected from the affiliated teaching hospitals of medical schools. Therefore, the representativeness of the survey results is limited. Second, confidentiality is an issue. The surveys were anonymous to protect the respondents’ rights. Third, the survey data are self-reported and thus are not externally valid. Fourth, although the significance of our research is obviously, some time has passed since the survey was implemented in 2006. This is due, in part, to the need to translate the findings into English. We believe it is necessary to update our study and findings now and we are setting out to do it.

## Conclusion

In summary, the results of this survey show that the utilization of global standards for PGME in evaluating the process and outcomes of PGME reform is conducive to timely identification and problem-solving, thus further enhancing medical education reform, the promotion of standardization and internationalization of PGME, and ultimately the overall improvement of the quality of PGME.

## Competing interests

The authors declare that they have no competing interests.

## Authors’ contributions

HB, DHZ, and TMZ planned, designed and conceived the study. DBX and ZJD drafted the manuscript. JSG contributed to the interpretation of the data and revised the manuscript. MNL performed statistical analyses. BZS and JZ piloted the survey. All authors read and approved the final manuscript.

## Pre-publication history

The pre-publication history for this paper can be accessed here:

http://www.biomedcentral.com/1472-6920/14/111/prepub
